# Evaluation of 3-Chlorobenzoate 1,2-Dioxygenase Inhibition by 2- and 4-Chlorobenzoate with a Cell-Based Technique

**DOI:** 10.3390/bios9030106

**Published:** 2019-09-05

**Authors:** Elena V. Emelyanova, Inna P. Solyanikova

**Affiliations:** Federal Research Center “Pushchino Biological Research Center of the Russian Academy of Sciences”, G.K. Skryabin Institute of Biochemistry and Physiology of Microorganisms of the Russian Academy of Sciences, 142290 Pushchino, Moscow Region, Russia; innas@ibpm.pushchino.ru

**Keywords:** actinobacterium *Rhodococcus opacus*, intact freshly harvested cells, nonmetabolized substrate, electrochemical reactor microbial sensor, dioxygenase inhibition by substrate analogues

## Abstract

The electrochemical reactor microbial sensor with the Clark oxygen electrode as the transducer was used for investigation of the competition between 3-chlorobenzoate (3-CBA) and its analogues, 2- and 4-chlorobenzoate (2-CBA and 4-CBA), for 3-chlorobenzoate-1,2-dioxygenase (3-CBDO) of *Rhodococcus opacus* 1CP cells. The change in respiration of freshly harvested *R. opacus* 1CP cells in response to 3-CBA served as an indicator of 3-CBDO activity. The results obtained confirmed inducibility of 3-CBDO. Sigmoidal dependency of the rate of the enzymatic reaction on the concentration of 3-CBA was obtained and positive kinetic cooperativity by a substrate was shown for 3-CBDO. The Hill concentration constant, *S_0.5_*, and the constant of catalytic activity, *V_max_*, were determined. Inhibition of the rate of enzymatic reaction by excess substrate, 3-CBA, was observed. Associative (competitive inhibition according to classic classification) and transient types of the 3-CBA-1,2-DO inhibition by 2-CBA and 4-CBA, respectively, were found. The kinetic parameters such as *S_0.5_^i^* and *V_max_^i^* were also estimated for 2-CBA and 4-CBA. The disappearance of the S-shape of the curve of the *V* versus *S* dependence for 3-CBDO in the presence of 4-CBA was assumed to imply that 4-chlorobenzoate had no capability to be catalytically transformed by 3-chlorobenzoate-1,2-dioxygenase of *Rhodococcus opacus* 1CP cells.

## 1. Introduction

Chlorobenzoates as herbicides are widely used in agriculture [1]. 3-chlorobenzoic acid (3-CBA) is also applied in industry as a raw material for fabrication of dyes, pharmaceutical preparations and fungicides and as a preserving agent for adhesive and dye. This compound can accumulate in the environment as a result of co-metabolism of polychlorobiphenyls and chlorotoluenes [2,3] and water chlorination. 3-CBA is, therefore, detected in industrial wastewaters, rivers and underground water [4].

For bioremediation of contaminated soils, soil microflora is activated and strains-destructors are introduced. Many bacterial species of genera *Pseudomonas* and *Rhodococcus* are able to break down xenobiotics including chlorobenzoates and chlorophenols, these species can be used as destructors [5,6,7,8]. Selection and introduction of new perspective strains-destructors are of current interest. In this context, it is important to know how xenobiotics affect the strain-destructor and how the activities of xenobiotic-metabolizing enzymes are altered.

In fact, all chloroaromatic compounds have a negative impact on living organisms [4,9]. It should be noted that with more substituents on the aromatic ring, the greater the resistance of the substance molecule to microbial degradation [10]. Degradation of the pollutants is sometimes impossible because of a narrow substrate specificity of the first enzyme in the degradative pathway.

A relatively narrow substrate specificity of benzoate 1,2-dioxygenase (BDO), that may initiate degradation of chlorobenzoates in *Acinetobacter calcoaceticus*, *Rhodococcus* sp. RHA1 and *Rhodococcus opacus* 1CP, leads to the negligible degradation of chlorobenzoates by this enzyme [11,12,13]. It has been shown that a narrow specific BDO of *Rhodococcus* sp. RHA1 may act both on benzoate and 3-chlorobenzoate but not on 2- and 4-chlorobenzoate (2-CBA, 4-CBA) [12].

It has been found that the complete destruction of 3-CBA by *Pseudomonas* sp. B13 began from the action of the enzyme, chlorobenzoate 1,2-dioxygenase (CBDO), on chlorobenzoate [1]. Metabolic pathways for the degradation of 3-CBA were compared in the representatives of the genus *Rhodococcus*, *R. opacus* 1CP and *R. opacus* 6a, being active destructors of phenol and 4-chlorophenol. It has been shown that particular features of substrate specificity of 3-chlorobenzoate-1,2-dioxygenase (3-CBA-1,2-DO or 3-CBDO), which initiated 3-CBA degradation, but not an enzyme pool of these strains were responsible for the metabolic pathway of 3-CBA degradation [14].

The activities of stable soluble enzymes of non-complex structures can be determined in cell-free extracts. Benzoate 1,2-dioxygenase is a bicomponent enzyme complex comprising reductase and oxidase; the latter component composes of two types of subunits, α and β [15,16]. It is impossible to identify BDO in the cell-free extract because of the loss of enzyme activity during destruction of microbial cells. For the determination of BDO activity, suspension of intact cells has to be used. A native active state of 3-chlorobenzoate-1,2-dioxygenase is not retained either after cell destruction. 3-chlorobenzoate-1,2-dioxygenase similar to BDO catalyzes the reaction in which oxygen is involved [17]. Thus, BDO and CBDO activities are measured in whole cells by the change in oxygen consumption by cells in the presence of the enzyme substrate: the rate of cell response to enzyme substrate [18]. The measurement is performed in suspension of intact freshly harvested microbial cells. Therefore, a reactor microbial biosensor with a Clark oxygen electrode as a transducer can be used [19] for determination of the cells’ response to a substrate. In this case, the suspension of microbial cells will generate responses to a substrate. Magnitudes of these responses will be proportional to activity of the enzyme.

The aim of the present study was to assess the competition between 3-CBA and its analogues (2-CBA and 4-CBA) for 3-CBDO, estimate the kinetic constants and determine the type of the enzyme inhibition by these analogues for *Rhodococcus opacus* 1CP using the electrochemical reactor sensor based on the intact cells of the studied culture.

## 2. Materials and Methods 

### 2.1. The Microorganism and Culture Conditions

The object of our study was Gram-positive non-spore-forming actinobacterium *Rhodococcus opacus* 1CP (DSM 46757 and VKM Ac-2638) isolated from the selective medium with 2,4-dichlorophenol. This culture was able to grow in media with aromatic compounds including benzoate as a sole source of carbon and energy [20].

The strain was stored as resting cells at +4 °C in a sterile buffer for 5 years. To activate cells and prepare inoculum, 10 μL of resting cell suspension was inoculated on the agarized Luria-Bertani (LB) medium (without 3-CBA) and incubated at +28 °C until germination.

Germinated cells were then washed out with 10 mL of the mineral medium of the following composition (g/L): Na_2_HPO_4_—0.73; KH_2_PO_4_—0.35; MgSO_4_ · 7H_2_O—0.1; NaHCO_3_—0.25; MnSO_4_—0.002; NH_4_NO_3_—0.75; FeSO_4_ · 7H_2_O—0.02. To obtain 3-CBA-grown *R. opacus* 1 CP cells, the suspension of germinated cells was inoculated into the mineral medium with 3-chlorobenzoate (50 mg/L) as the sole source of carbon and energy. *R. opacus* 1 CP cells were grown in 750-mL Erlenmeyer flasks with 200 mL of the medium at +29 °C on a rotary shaker (n = 220 rpm). During culture cultivation 3-CBA was added once more into the medium (100 mg/L) after consumption of the substrate.

### 2.2. Biosensor Determination of 3-Chlorobenzoate-1,2-Dioxygenase Activity

The activity of 3-chlorobenzoate-1,2-dioxygenase was determined as the change in respiration of intact freshly harvested 3-CBA-grown cells of actinobacterium in response to the enzyme substrate (the rate of cells response to a substrate, i.e., the rate of enzymatic reaction) using the electrochemical reactor microbial sensor.

For determination of the activity of 3-chlorobenzoate-1,2-dioxygenase, 3-CBA-grown *R. opacus* 1 CP cells were separated by centrifugation (7000 g, 10 min, +4 °C), washed twice with 50-mM Tris-HCl, pH 8.0, suspended in the same buffer and immediately used for analysis.

The measurements were performed in an air-saturated 50-mM Tris-HCl buffer, with a pH of 8.0, at room temperature in a 5-mL open cell equipped with a mixer. When basal cell respiration level was stabilized, 3-CBA was injected into the cell and the rate of oxygen concentration change was measured using a Clark oxygen electrode. The recorded signal (the rate of cell response to 3-CBA) reflected the rate of the enzymatic reaction of 3-CBDO with the substrate. The rate unit was pA/s: 1 pA/s ~ 0.153 μg O_2_/(L s).

The oxygen electrode was equipped with an “Ingold 531 O_2_ amplifier” (Instrumentation Laboratory (MI), Milan, Italy). The signal was recorded with a two-coordinate “XY Recorder-4103” (Laboratorni Přistroje, Praha, Czech Republic).

### 2.3. Evaluation of 3-CBDO Inhibition by 2- and 4-CBA

To reveal the competition between 3-chlorobenzoate and its analogues, 2-chlorobenzoate and 4-chlorobenzoate, for 3-CBDO, the substrate of 3-CBA-1,2-DO (3-CBA) or 3-CBA simultaneously with 2-CBA or 4-CBA were injected into the cell with suspension of *R. opacus* 1CP cells. The change in oxygen concentration was then registered. The rates of change of oxygen consumption by *R. opacus* 1CP cells at a separate (only 3-CBA) or simultaneous (3-CBA with 2-CBA or 3-CBA with 4-CBA) injection of two compounds were compared.

To determine the type of inhibition, curves of the dependence of the rate of enzymatic reaction (*V*) on 3-CBA concentration (*S*) in the presence and absence of inhibitors (2-CBA or 4-CBA) were plotted in *V-S* and bireciprocal coordinates. The type of 3-CBA-1,2-BDO inhibition was determined by interposition of *V* vs. *S* curves and the magnitudes of kinetic constants (*V_max_* and *S_0.5_*) using the method of vector representation of enzymatic reactions [21].

#### Statistics

The measurements were taken in triplicate in two independent series of experiments. The presented results reflected averaged values. Statistical data analysis was carried out using a Student’s *t*-test (*p* < 0.05).

## 3. Results and Discussion

### 3.1. Estimation of 3-CBA-1,2-DO Activity

The rate of enzymatic reaction catalyzed by 3-chlorobenzoate-1,2-dioxygenase was measured indirectly by the change of *R. opacus* 1CP cells respiration in the presence of 3-CBDO substrates (the rate of response to a substrate). The change in the respiration rate of freshly harvested *R. opacus* 1CP cells in the presence of 3-CBA was an indicator of the presence of 3-chlorobenzoate-1,2-dioxygenase. The change in intensity (rate) of respiration of freshly harvested cells in response to 3-CBA was a complex response of all of the cells but not of the enzyme (3-CBDO) of the cells. Thus, the results obtained were indirect estimates of 3-CBA-1,2-DO activity.

**Figure 1:**
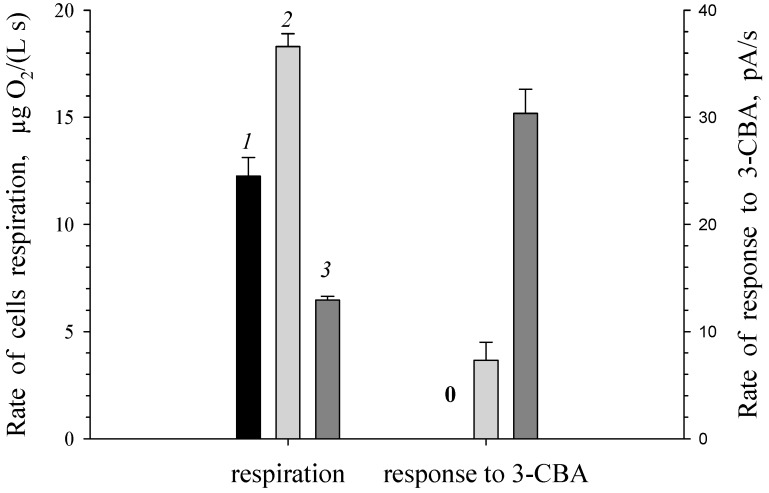
Comparison of respiration and response to 3-CBA for suspensions of *R. opacus* 1CP cells. (*1*) LB-grown freshly harvested cells were suspended in buffer. (*2*) Luria-Bertani-grown freshly harvested cells were induced by 3-CBA in buffer. (*3*) 3-CBA-grown freshly harvested cells were suspended in buffer. Symbols: **0** denotes the rate of response to 3-CBA was 0 pA/s.

It is known, that BDO of *R. opacus* 1CP cells is an inducible enzyme. By analogy with BDO, it is expected that 3-CBDO is also an inducible enzyme. Responses to 3-CBA for *R. opacus* 1CP cells, grown without 3-CBA (LB-grown cells) and in the presence of 3-CBA (3-CBA-grown cells), were investigated (Figure 1). When a substrate of 3-chlorobenzoate-1,2-dioxygenase (3-CBA) was absent in growth medium, *R. opacus* 1CP cells contained only the basal (low) amount of the enzyme. No 3-chlorobenzoate-1,2-dioxygenase activity was detected by response to 3-chlorobenzoate for a suspension of freshly harvested LB-grown cells. Response to 3-CBA was obtained only for 3-CBA-grown and induced by 3-CBA cells indicating inducibility of 3-chlorobenzoate-1,2-dioxygenase. When *R. opacus* 1CP was grown in the medium with 3-CBA as the sole carbon and energy source, the response of cells to this substrate was higher than that for cells induced by 3CBA in non-growth conditions (in buffer solution).

**Figure 2:**
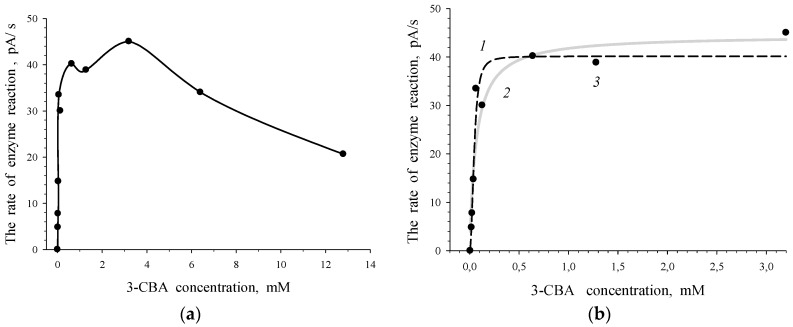
Dependence of the rate of enzymatic reaction (*V*, pA/s), catalyzed by 3-CBA-1,2-DO of *R. opacus* 1CP cells grown in the medium with 3-CBA, on the initial 3-chlorobenzoate concentration (*S*, mM): (**a**) experimental data curve; (**b**) curves plotted by the Sigma Plot program using experimental data (*3*). Calculated curves: a sigmoidal curve described by the Hill equation (*1*) and a hyperbolic curve of Michaelis–Menten (*2*).

To evaluate the 3-chlorobenzoate-1,2-dioxygenase activity, *R. opacus* 1CP cells were grown in the medium with 3-CBA as the sole source of carbon and energy. The rate of response of intact freshly harvested cells (the suspension of freshly harvested cells of actinobacterium in 50 mM Tris/HCl buffer, pH 8.0) to 3-chlorobenzoate was determined. The curve of the dependence of the rate of enzyme reaction on 3-CBA concentration was obtained for *R. opacus* 1CP 3-CBA-grown cells (Figure 2a). The obtained curve depicted the dependence of the rate of reaction between 3-chlorobenzoate-1,2-dioxygenase of 1CP cells and the substrate on initial concentration of 3-CBA (substrate of 3-CBA-1,2-DO). For 3-CBA-grown cells, the dependency of the rate of enzyme reaction on 3-CBA concentration could be described more accurately (0.9757 > 0.9526) using the Hill equation (sigmoid plot) rather than Michaelis–Menten equation (hyperbolic plot). The S-shape (curve *1*) and hyperbolic (curve *2*) curves, plotted with experimental data (points *3*), are shown in Figure 2b. An S-shaped type of the dependence of *V* on *S* for 3-CBA-1,2-DO of *R. opacus* 1CP confirmed deviation from the Michaelis–Menten kinetics.

A sigmoidal dependency of the rate of enzymatic reaction on the concentration of a substrate or metabolite-regulator and subunit nature of enzyme molecule, composed of several identical monomers (subunits), are the characteristics of allosteric enzymes [22]. Benzoate-1,2-dioxygenase of *R. opacus* 1CP is an allosteric enzyme. It consists of two different subunits. The sigmoidal dependency of *V* on *S* of benzoate and positive kinetic cooperativity by a substrate were observed for BDO of *R. opacus* 1CP cells [23]. It might be expected that the dependence of *V* on *S* for 3-CBA-1,2-DO of this culture would be sigmoidal. For calculation of the kinetic constants of 3-CBA-1,2-DO, experimental data obtained for enzyme-containing cells were statistically processed by the Sigma Plot program using Hill’s empirical equation:

*V* = *V*_max_·*S^n^/*(*S*_0.5_*^n^* + *S^n^*), (1)

where

*V_max_* was the maximum rate of the enzymatic reaction (maximal magnitude of the rate when *S* → ∞; a constant of catalytic activity);
*S_0.5_* was the substrate concentration (*S*) when *V* = 0.5*V_max_* (a Hill concentration constant);
*n* was a Hill coefficient.

The Hill coefficient (*n*) had no physical sense. It implied that kinetics of the process for 3-CBA-1,2-DO (dependency of *V* on concentration of 3-CBA) deviated from the classic hyperbolic saturation kinetics, a typical Michaelis–Menten kinetics. The magnitude of *n* may be < 1 or > 1 that points to “negative” or “positive kinetic cooperativity” by a substrate. In this study, the Hill coefficient (*n*) was higher than one (2.36) for 3-CBA-grown *R. opacus* 1CP cells, which contained 3-CBA-1,2-DO. This fact was in accordance with the deviation of the *V* vs. *S* dependency from the classic hyperbolic dependency for 3-CBA-1,2-DO of 3-CBA-grown cells. The Hill concentration constant, *S_0.5_*, was 45.3 ± 6.3 µM, and the constant of catalytic activity, *V_max_*, was 40.2 ± 2.4 pA/s.

**Figure 3:**
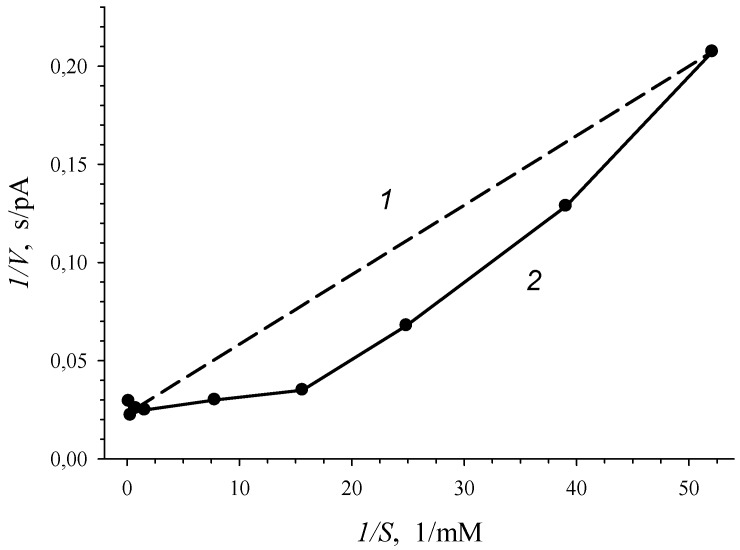
Dependence of the rate of enzymatic reaction (*V*, pA/s), catalyzed by 3-CBA-1,2-DO of 3-CBA-grown *R. opacus* 1CP cells, on the initial 3-chlorobenzoate concentration (*S*, mM) in bireciprocal *1/V-1/S* (*2*) coordinates. Line (*1*) is a hyperbolic dependence in bireciprocal *1/V-1/S* coordinates.

Graphical confirmation of the S-shape of dependence of *V* on *S*, which was shown in Figure 2b, is a plot in Figure 3, where experimental data were given in bireciprocal *1/V* vs. *1/S* coordinates. Concavity of a curve of *V* vs. *S* plotted in bireciprocal coordinates (deviation of the solid line from the dash line in Figure 3) for allosteric enzyme pointed to “positive kinetic cooperativity” by a substrate for the enzyme of *R. opacus* 1CP cells. Therefore, positive kinetic cooperativity by a substrate was shown for 3-CBDO of 3-CBA-grown *R. opacus* 1CP cells: the affinity for substrate increased as the substrate bound with active sites on the enzyme molecule.

Positive kinetic cooperativity by a substrate for allosteric enzymes occurs because of the interaction between substrate-binding sites in the enzyme molecule [22]. In addition to catalytic sites a molecule of the allosteric enzyme may contain regulatory sites for a substrate. When the substrate binds with the regulatory site, it does not undergo catalytic conversion, but influences the catalytic efficiency of active sites. Inhibition of the rate of the enzymatic reaction by excess substrate (the plot of the dependence of *V* vs. *S* in Figure 2a), that could be of allosteric nature, was observed for 3-CBA-1,2-DO of 3-CBA-grown cells. The maximum on the curve of the dependence of *V* on *S* could be the result of the interaction between 3-CBA-binding sites in the molecule of 3-CBA-1,2-DO. Obviously, the catalytic efficiency of active sites of the enzyme decreased because of interaction of active sites with regulatory sites for a substrate that was observed in the study of Katsumata and Goldman [24].

### 3.2. 3-Chlorobenzoate-1,2-Dioxygenase Inhibition by 2- and 4-Chlorobenzoate for R. opacus 1CP 3-CBA-Grown Cells

3-chlorobenzoate-1,2-dioxygenase of *R. opacus* 1CP, which was studied by Solyanikova et al. [14], was the enzyme with narrow substrate specificity. It was yet unclear, whether such specificity was the result of extremely high selectivity of 3-CBA-1,2-DO and substituted benzoates did not bind to active sites on the enzyme. Or whether these substrates bound to active sites on the enzyme, but reaction did not occur. To clarify this, the competition between 3-chlorobenzoate and its analogues, 4-CBA and 2-CBA, for 3-CBA-1,2-DO was estimated.

**Figure 4:**
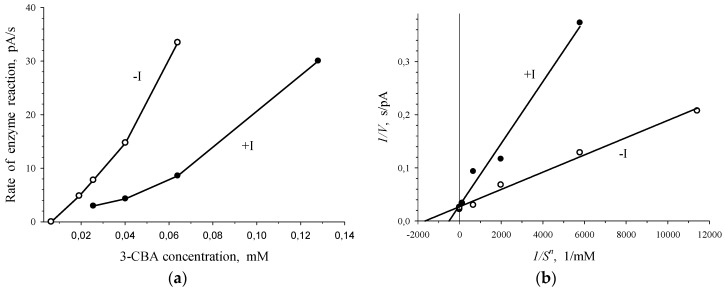
Graphical evaluation of the type of inhibition of *R. opacus* 1CP 3-CBA-1,2-DO by 2-chlorobenzoate. Dependence of *V* on *S* in *V-S* coordinates (**a**) and in bireciprocal coordinates (**b**) in the absence (-*I*) and presence (+*I*) of the inhibitor of 1CP 3-CBA-1,2-DO: 0.8 mM 2-CBA.

To determine the type of enzyme inhibition by 2-chlorobenzoate, plots of the *V* vs. *S* dependence for 3-chlorobenzoate-1,2-dioxygenase in the absence and presence of 2-CBA were drawn in *V-S* and *1/V-1/S^n^* coordinates (Figure 4). In the presence of 2-CBA, the catalytic constant of the process, *V_max_*, did not change, but the reduction of the Hill concentration constant, *S_0.5_*, was observed. In the presence of a substrate analogue of 3-CBA (2-CBA) the next ratios of kinetic parameters were obtained: *V_max_^0^* = *V_max_^i^*; *S_0.5_^0^* < *S_0.5_^i^*, where *V_max_^0^*, *S_0.5_^0^* and *V_max_^i^*, *S_0.5_^i^* were kinetic constants of the enzyme in the absence and presence of 2-CBA, respectively. Based on the aforementioned ratios, the conclusion was made on associative (competitive inhibition according to classic classification) inhibition [21] of 3-chlorobenzoate-1,2-dioxygenase by 2-chlorobenzoate.

**Figure 5:**
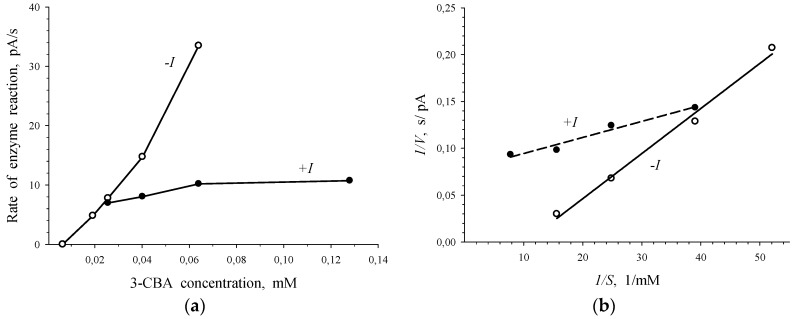
Competition between 3-CBA and 4-CBA for 3-CBA-1,2-DO of *R. opacus* 1CP cells. Dependence of *V* on *S* in *V-S* coordinates (**a**) and bireciprocal coordinates (**b**) in the absence of 4-CBA (only 3-CBA injection: - *I*) and presence of 0.8 mM 4-CBA (injection of 3-CBA with 4-CBA: +*I*).

Plots presented in Figure 5 point to the competition between 3-CBA and 4-CBA for 3-CBA-1,2-DO of *R. opacus* 1CP cells. Simultaneous injection of 3-CBA (concentrations lower than 0.025 mM) with 0.8 mM of 4-CBA led to activation of the enzymatic process (Figure 5a). However, at 3-CBA concentration that was higher than 0.025 mM, an addition of 0.8 mM of 4-CBA declined catalytic activity. Studied sections of the curve of the *V* vs. *S* dependence, depicted in Figure 5a, were plotted in Lineweaver–Burk coordinates, bireciprocal *1/V-1/S* coordinates (Figure 5b). In the presence of only 3-CBA (*-I*) after linear extrapolation, the line plotted in *1/V-1/S* coordinates will have intersected the *1/S* axe in the region of positive magnitudes of *1/S*. It is characteristic of the S-shape dependency of *V* on *S,* which is shown for the dependence of the rate of enzyme (3-CBA-1,2-DO) reaction on 3-CBA concentration (Figure 2 and Figure 3). After linear extrapolation of the line plotted for a case of the simultaneous presence of 3-CBA and 4-CBA, the point of line intersection with *1/S* axe will have been in the region of negative magnitudes of *1/S*. It points to the hyperbolic dependency of *V* on *S*. In the presence of 4-CBA (a substrate analogue of 3-CBA), a decrease in the catalytic activity of the process (catalytic constant *V_max_*) and a reduction in the Hill concentration constant (*S_0.5_*), which was a graphic analogue of *K_m_*, were found.

Thus, simultaneous injection of 4-CBA and 3-CBA resulted in disappearance of the S-shape of the curve of *V* vs. *S* dependency for *R. opacus* 1CP cells. Furthermore, activation of the process was observed at low concentrations (<0.025 mM) of the substrate, 3-CBA. Concavity of a curve for the dependence of the rate of enzymatic reaction catalyzed by 3-CBA-1,2-DO of *R. opacus* 1CP cells on the substrate, indicating positive kinetic cooperativity by a substrate, has already been shown in Figure 3. A specific feature of allosteric enzymes with positive kinetic cooperativity by a substrate is known from literature. If a substrate analogue without capability to be catalytically converted was injected into the reaction system, it led to significant changes. For example, the S-shape of the curve of the *V* vs. *S* dependence disappeared, and Wittenberger and Fulco [25] observed activation of an allosteric enzyme at low concentrations of the substrate. Apparently, 4-chlorobenzoate, which is a substrate analogue of 3-CBA, is incapable of being catalytically transformed by 3-chlorobenzoate-1,2-dioxygenase.

It is known, that the allosteric enzyme did not lose its ability to catalyze the reaction at spatial isolation of the catalytic and the regulatory sites of the enzyme, but in this case kinetic cooperativity by the substrate was not found. Probably, in our study 4-CBA acted as a substrate analogue of 3-CBA (the analogue with no capacity for catalytic transformation by 3-chlorobenzoate 1,2-dioxygenase) bound with regulatory sites for the substrate and was not catalytically transformed by 3-CBA-1,2-DO of *R. opacus* 1CP cells. This led to the differences in kinetics for 2-CBA and 4-CBA that are shown in the Figures: it is seen that Figure 5b differs from Figure 4b.

The difference in the action of 4-CBA from that of 2-CBA was that in the presence of 4-CBA a reduction of catalytic activity of the process was observed: the magnitude of *V_max_* altered from 40.2 ± 2.4 pA/s to 12.8 ± 0.9 pA/s. However, the action of 4-CBA was similar to that of 2-CBA that the decrease of Hill concentration constant (*S_0.5_*) was observed. *S_0.5_* slowed down to 21.3 ± 5.3 µM. Comparison of kinetic constants in the absence (*V_max_^0^*, *S_0.5_^0^*) and presence (*V_max_^i^*, *S_0.5_^i^*) of 4-CBA led to the results as follows: *V_max_^0^* > *V_max_^i^* (40.2 pA/s > 12.8 pA/s) and *S_0.5_^0^* > *S*_0.5_^i^ (45.3 µM > 21.3 µM). It became clear that in the presence of 4-CBA, transient inhibition was observed: transition from activation of the process at low substrate concentrations (below 0.025 mM) to inhibition at 3-CBA concentrations above 0.025 mM.

## 4. Conclusions

Associative (competitive inhibition according to classic classification) and transient types of the 3-CBA-1,2-DO inhibition by 2-CBA and 4-CBA, respectively, were found using the electrochemical reactor sensor based on the intact cells of *R. opacus* 1CP. Based on the results obtained from our study we assumed that 2-CBA bound to the active sites of 3-CBA-1,2-DO and competed with 3-CBA for the enzyme. In contrast 4-CBA could bind to the regulatory sites of the enzyme, 3-chlorobenzoate-1,2-dioxygenase, altering kinetics of the enzyme and this substrate could not be metabolized by 3-CBA-grown *R. opacus* 1CP cells. The latter assumption needs to be proven with further research.
